# Supplementary material for: Defining healthcare never events to effect system change: A protocol for systematic review
Source: PLoS One. 2022 Dec 15;17(12):e0279113. doi: 10.1371/journal.pone.0279113 (PMC9754204; doi:10.1371/journal.pone.0279113)
Supplement: S2 Appendix — (DOCX) [file pone.0279113.s002.docx]

# Appendix 2: Search Strategies

**Medline**

1. Patients/

2. (patient* or client* or user or users or consumer*).ti,ab,kw,kf.

3. (safety or safeties or harm*).ti,ab,kw,kf.

4. 1 or 2

5. 3 and 4

6. Patient Safety/ or Patient Harm/ or Accident Prevention/

7. (open adj2 disclosure*).ti,ab,kw,kf.

8. (mandatory adj2 disclosure*).ti,ab,kf,kw.

9. "duty of candor".ti,ab,kw,kf.

10. or/5-9

11. ((critical* or sentinel or serious) adj1 (incident* or event* or harm or error*)).ti,ab,kw,kf.

12. ((clinical or surg*) adj2 error*).ti,ab,kf,kw.

13. (never adj1 event*).ti,ab,kf,kw.

14. "serious patient safety incident".ti,ab,kw,kf.

15. "patient safety learning system".ti,ab,kw,kf.

16. "critical incident reporting system".ti,ab,kf,kw.

17. (medic* adj2 mistake*).ti,ab,kf,kw.

18. (wrong adj3 (error* or surger*)).ti,ab,kw,kf.

19. or/11-18

20. 10 and 19

21. limit 20 to yr="2001 -Current"

**CENTRAL**

1. exp patients/

2. (patient* or client* or user or users or consumer*).ti,ab.

3. (safety or safeties or harm*).ti,ab.

4. 1 or 2

5. 3 and 4

6. patient safety/

7. (open adj2 disclosure*).ti,ab.

8. (mandatory adj2 disclosure*).ti,ab.

9. "duty of candor".ti,ab.

10. or/6-9

11. ((critical* or sentinel or serious) adj1 (incident* or event* or harm or error*)).ti,ab.

12. ((clinical or surg*) adj2 error*).ti,ab.

13. (never adj1 event*).ti,ab.

14. "serious patient safety incident".ti,ab.

15. "patient safety learning system".ti,ab.

16. "critical incident reporting system".ti,ab.

17. (medic* adj2 mistake*).ti,ab.

18. (wrong adj3 (error* or surger*)).ti,ab.

19. or/11-18

20. 10 and 19

21. limit 20 to yr="2001 -Current"

**Embase**

1. patient/

2. (patient* or client* or user or users or consumer*).ti,ab,kw,kf.

3. (safety or safeties or harm*).ti,ab,kw,kf.

4. 1 or 2

5. 3 and 4

6. exp patient safety/

7. accident prevention/

8. (open adj2 disclosure*).ti,ab,kw,kf.

9. (mandatory adj2 disclosure*).ti,ab,kf,kw.

10. "duty of candor".ti,ab,kw,kf.

11. or/5-10

12. ((critical* or sentinel or serious) adj1 (incident* or event* or harm or error*)).ti,ab,kw,kf.

13. ((clinical or surg*) adj2 error*).ti,ab,kf,kw.

14. (never adj1 event*).ti,ab,kf,kw.

15. "serious patient safety incident".ti,ab,kw,kf.

16. "patient safety learning system".ti,ab,kw,kf.

17. "critical incident reporting system".ti,ab,kf,kw.

18. (medic* adj2 mistake*).ti,ab,kf,kw.

19. (wrong adj3 (error* or surger*)).ti,ab,kw,kf.

20. or/12-19

21. 11 and 20

22. limit 21 to yr="2001 -Current"

**APA PsycINFO**

1. exp patients/

2. (patient* or client* or user or users or consumer*).ti,ab.

3. (safety or safeties or harm*).ti,ab.

4. 1 or 2

5. 3 and 4

6. patient safety/

7. (open adj2 disclosure*).ti,ab.

8. (mandatory adj2 disclosure*).ti,ab.

9. "duty of candor".ti,ab.

10. or/6-9

11. ((critical* or sentinel or serious) adj1 (incident* or event* or harm or error*)).ti,ab.

12. ((clinical or surg*) adj2 error*).ti,ab.

13. (never adj1 event*).ti,ab.

14. "serious patient safety incident".ti,ab.

15. "patient safety learning system".ti,ab.

16. "critical incident reporting system".ti,ab.

17. (medic* adj2 mistake*).ti,ab.

18. (wrong adj3 (error* or surger*)).ti,ab.

19. or/11-18

20. 10 and 19

21. limit 20 to yr="2001 -Current"

**CINAHL**

| **Search Terms** | **Search Options** |
| --- | --- |
| S16 | S7 AND S15 |
| S15 | S8 OR S9 OR S10 OR S11 OR S12 OR S13 OR S14 |
| S14 | TI ( (wrong N3 (error* or surger*)) ) OR AB ( (wrong N3 (error* or surger*)) ) |
| S13 | TI (medic* N2 mistake*) OR AB (medic* N2 mistake*) |
| S12 | TI (never N1 event*) OR AB (never adj1 event*) |
| S11 | TI ( ((clinical or surg*) N2 error*) ) OR AB ( ((clinical or surg*) N2 error*) ) |
| S10 | TI ( ((critical* or sentinel or serious) N1 (incident* or event* or harm or error*)) ) OR AB ( ((critical* or sentinel or serious) N1 (incident* or event* or harm or error*)) ) |
| S9 | (MH "Sentinel Event") |
| S8 | (MH "Wrong Site Surgery") |
| S7 | S5 OR S6 |
| S6 | (MH "Patient Safety") |
| S5 | S3 AND S4 |
| S4 | S1 OR S2 |
| S3 | TI ( (safety or safeties or harm*) ) OR AB ( (safety or safeties or harm*) ) |
| S2 | TI ( (patient* or client* or user or users or consumer*) ) OR AB ( (patient* or client* or user or users or consumer*) ) |
| S1 | (MH "Patients+") |
