# Supplementary material for: Defining healthcare never events to effect system change: A protocol for systematic review
Source: PLoS One. 2022 Dec 15;17(12):e0279113. doi: 10.1371/journal.pone.0279113 (PMC9754204; doi:10.1371/journal.pone.0279113)
Supplement: S3 Appendix — (DOCX) [file pone.0279113.s003.docx]

# Appendix 3: Preliminary Data Collection Form

| **Item** | **Options (if applicable)** |
| --- | --- |
| Title |  |
| Authors |  |
| Journal |  |
| Year |  |
| Country |  |
| Type of organization | National  Regional  Individual (specify)  Other (specify) Unknown |
| Type of article | Peer-reviewed Conference abstract Grey-lit (non-policy) Policy Other (specify) |
| Type of articles - other specify |  |
| Study design | RCT Cohort Case-control Quasi-experimental (incl. quality improvement) Descriptive (incl. Root Cause Analysis) Narrative summary Editorial or opinion Other (specify) |
| Medical specialty |  |
| Does the paper use existing NE framework(s)? | Y/N |
| List the framework(s) used |  |
| For frameworks which have more than 1 version, list the year(s) of the one(s) used |  |
| If article discusses "wrong site", do they mean any wrong (i.e. body part/patient/procedure), just the wrong body part, or specifically the wrong spinal / finger level, or some combination thereof? |  |
| If framework includes "foreign body", list which events are "foreign bodies" |  |

| **List the never events** | **For each event, identify its preventability (entirely, not entirely, unclear)** | **For each event, identify if it is currently in use, proposed, or retired** |
| --- | --- | --- |
|  |  |  |
|  |  |  |
